# Implications of Gene Inheritance Patterns on the Heterosis of Abdominal Fat Deposition in Chickens

**DOI:** 10.3390/genes10100824

**Published:** 2019-10-18

**Authors:** Chunning Mai, Chaoliang Wen, Congjiao Sun, Zhiyuan Xu, Sirui Chen, Ning Yang

**Affiliations:** 1 Department of Animal Genetics and Breeding, College of Animal Science and Technology, China Agricultural University, Beijing 100193, China; maicn@cau.edu.cn (C.M.); clwen@cau.edu.cn (C.W.); cjsun@cau.edu.cn (C.S.); forward-xu@hotmail.com (Z.X.); csr@cau.edu.cn (S.C.); 2 National Engineering Laboratory for Animal Breeding, and Key Laboratory of Animal Genetics, Breeding and Reproduction, Ministry of Agriculture and Rural Affairs, China Agricultural University, Beijing 100193, China

**Keywords:** heterosis, abdominal fat deposition, gene expression patterns, over-dominant genes, high-parent dominant genes, chicken

## Abstract

Heterosis, a phenomenon characterized by the superior performance of hybrid individuals relative to their parents, has been widely utilized in livestock and crop breeding, while the underlying genetic basis remains elusive in chickens. Here, we performed a reciprocal crossing experiment with broiler and layer chickens and conducted RNA sequencing on liver tissues for reciprocal crosses and their parental lines to identify inheritance patterns of gene expression. Our results showed that heterosis of the abdominal fat percentage was 69.28%–154.71% in reciprocal crosses. Over-dominant genes of reciprocal crosses were significantly enriched in three biological pathways, namely, butanoate metabolism, the synthesis and degradation of ketone bodies, and valine, leucine, and isoleucine degradation. Among these shared over-dominant genes, we found that a lipid-related gene, *HMGCL,* was enriched in these pathways. Furthermore, we validated this gene as over-dominant using qRT-PCR. Although no shared significant pathway was detected in the high-parent dominant genes of reciprocal crosses, high-parent dominant gene expression was the major gene inheritance pattern in reciprocal crosses and we could not exclude the effect of high-parent dominant genes. These findings suggest that non-additive genes play important roles in the heterosis of important traits in chickens and have important implications regarding our understanding of heterosis.

## 1. Introduction

Heterosis or, more narrowly, hybrid vigor refers to the phenomenon in which progenies exhibit greater biomass, speed of development, or fertility than their parents [1]. Plant and animal breeders exploit heterosis by mating two different pure-bred lines with certain desirable traits, and have driven unprecedented improvements in performance or viability in crops and livestock [2,3]. However, heterosis does not always imply superiority, as it depends on the trait’s biological significance and production preference. Several studies demonstrated that maize and wheat exhibiting heterosis for height had weaker lodging resistance than shorter individuals [4,5]. It is also well understood that a greater cocoon shell thickness affects the hatching of silkworms [6,7].

Broilers and layers are two distinct, modern, commercial chicken types, which are selected for body weight and egg production, respectively [8]. Abundant evidence has reported the existence of heterosis for abdominal fat deposition in crossing progenies when parental lines have distinct genetic differences [9,10,11]. Excessive fat deposition is an unfavorable trait in chickens since it reduces feed efficiency, reduces the yield of edible carcass, and affects consumer acceptance [12]. In mammals, de novo fatty acid synthesis occurs mainly in adipose, liver, and mammary tissues [13]. In chickens, 95% of fatty acids are synthesized in the liver, which plays an important role in fat deposition and metabolism [14].

Dominance and over-dominance are two classic hypotheses that have been proposed to explain heterosis [15,16]. The dominance hypothesis suggests that deleterious alleles at different loci in homozygous parents are complemented in heterozygous progenies [17]. The over-dominance hypothesis states that the improved performance of progenies relative to their parents is a consequence of allelic interactions at heterozygous loci that outperform the homozygous state [18]. More recently, the two classic hypotheses were reinterpreted in terms of non-additivity, which was further categorized into high-parent dominance, low-parent dominance, over-dominance, and under-dominance [19]. 

With the help of high-throughput sequencing technology, RNA sequencing provides an opportunity to analyze the relationship between heterosis and differences in gene expression of reciprocal crosses and their parents [20,21]. In this study, two distinct chicken breeds, Cornish (C) and Rhode Island White (R), which were selected for growth and egg production, respectively, were used as parents to produce purebred (CC and RR) and crossbred (CR and RC) progenies. Liver tissues were employed for RNA sequencing to identify transcriptomic differences in gene expression for reciprocal crosses and their parental lines. The objective of this study was to provide insight into the genetic basis of the heterosis of abdominal fat deposition in chickens.

## 2. Materials and Methods

### 2.1. Experimental Populations and Phenotypic Measurement

All procedures were performed according to guidelines established by the Animal Care and Use Committee of China Agricultural University (approval No. SYXK [Jing] 2013-0013).

Two chicken breeds, Cornish and Rhode Island White, were used as parents in this study to produce purebred and crossbred progenies. Cornish chickens originated from Cornwall, England, and are widely used in the meat industry. Rhode Island White chickens originated from Rhode Island, US; this breed is dual-purpose, suitable for both meat and egg production. The birds used in our study were kept at the farm of Beijing Huadu Yukou Poultry Industry Co., Ltd. of China. The C line was selected for body weight after 42 days for 7 generations, and the R line was selected mainly for total egg production at 44 weeks of age for 15 generations. In total, 10 males and 120 females from the 8th generation of the C line and 10 males and 80 females from the 16th generation of the R line were selected as parents and reared in individual cages in the same poultry facility. Each male of C and R was mated with six C and four R females by artificial insemination, and eggs were collected for ten days. A total of 347 female and 285 male chicks were hatched on the same day and reared in cages with food and water ad libitum.

Given that modern broilers are mostly marketed at six weeks of age, 41 females (8, 8, 10, and 15 for CC, RR, CR, and RC, respectively) and 44 males (10, 9, 15, and 10 for CC, RR, CR, and RC, respectively) from different half-sib families were randomly selected to be euthanized by cervical dislocation and measured for abdominal fat content (surrounding the gizzard, cloaca, and adjacent abdominal muscles) at six weeks of age (Supplementary Figure S1). The abdominal fat percentage (AFP) was calculated as the percentage of abdominal fat weight of the total body weight at six weeks of age. Heterosis (as a percentage, H%) of the AFP (%) was calculated according to the following equation:

(1) $$H\%=\frac{\bar{F_{1}}-\left(\bar{P_{1}}+\bar{P_{2}}\right)/2}{\left(\bar{P_{1}}+\bar{P_{2}}\right)/2}\;\times 100\%$$

where $\bar{F_{1}}$, $\bar{P_{1}}$, and $\bar{P_{2}}$ are the average phenotypes of the F1 reciprocal crosses and their two parental lines, respectively. Student’s t-value was calculated following the formula of Wu et al. [22] to evaluate the significance of H%:

(2) $$t=\;\frac{H\%}{2\sqrt{\frac{\sum {\left(F_{1i}-\bar{F_{1}}\right)}^{2}}{n_{1}-1}}\;/\;\left[\left(\bar{P_{1}}+\bar{P_{2}}\right)\times \sqrt{n_{1}}\;\right]}$$

where $F_{1i}\;$ is the phenotype of chicken i from RC or CR and $n_{1}\;$ is the number of chickens in RC or CR. Based on the t-value and degrees of freedom, we obtained the p-value using the pt function in the R project (https://www.r-project.org/). H% was considered significant if the *p*-value was <0.05, and a *p*-value of <0.01 was considered extremely significant.

### 2.2. Sample Collection, RNA Extraction, and RNA Sequencing

Six female offspring from four to six half-sibling families of each of the groups, except for RC (n = 5), were randomly selected for sample collection. Six liver tissues were collected from RR, CR, and CC groups and five liver tissues were obtained from the RC group. Samples were immediately placed in liquid nitrogen and stored at −80 °C until RNA extraction.

TRIzol® Reagent (Invitrogen, Carlsbad, CA, USA) was used for total RNA extraction. To ensure successful RNA isolation, the total RNA was detected by 1% agarose gel electrophoresis. The RNA integrity number (RIN) was determined using the RNA Nano 6000 Assay Kit and the Bioanalyzer 2100 system (Agilent Technologies, Santa Clara, CA, USA). The RNA concentration was evaluated using the Qubit® RNA Assay Kit and the Qubit® 2.0 Flurometer (Life Technologies, Grand Island, NY, USA). The RNA purity was assessed using a NanoPhotometer® spectrophotometer (IMPLEN, Westlake Village, CA, USA). The NEBNext® UltraTM RNA Library Prep Kit (Illumina, San Diego, CA, USA) was used for RNA-seq library construction according to the manufacturer’s guide. The mRNA-seq libraries were sequenced on an Illumina Hiseq X Ten platform.

### 2.3. Differentially Expressed Gene Identification

The chicken reference genome (galGal5, fasta format) and annotation files (gtf format) were downloaded from the Ensemble database (ftp://ftp.ensembl.org/pub/release-91/). High-quality reads were obtained from raw reads by excluding reads with more than 50% low-quality bases (Qphred ≤ 20). Hisat2 (v2.0.5) was used for aligning high-quality reads to the reference genome [23]. The mapped reads of each sample were assembled by StringTie (v.1.3.3b) [24], and FPKM values were extracted from the StringTie outputs. The genes with an average FPKM of <1 were removed to enhance the statistical power for differentially expressed genes (DEGs). The DESeq2 package (v.1.16.1) [25] was used for differential expression analysis between the two purebred lines (CC vs. RR) and between reciprocal crosses and purebred lines (CR vs. CC, CR vs. RR, RC vs. CC, and RC vs. RR). Genes with an adjusted *p*-value of < 0.05 (BH multiple test correction) were considered to be differentially expressed genes.

### 2.4. Inheritance Mode Classification

Gene expression modes were divided into 12 types (I, II, III, IV, V, VI, VII, VIII, IX, X, XI, and XII; for details, see Table S1) according to Swanson-Wagner et al. [19] and Rapp et al. [26]. DEGs were further classified into additivity, high-parent dominance, low-parent dominance, over-dominance, and under-dominance based on the level of gene expression exhibited by reciprocal crosses and parental lines. Additivity (I, XII) represented that the gene expression was significantly different between the two parental lines (CC and RR, adjusted *p*-value < 0.05) and that the gene expression of reciprocal crosses (CR or RC) was not significantly different to the mean of their parental lines (1/2(CC + RR)). Gene expression in CR or RC that was not significantly different from one parental line but significantly higher than the other parental line was regarded as high-parent dominance (II and IV). Low-parent dominance (IX and XI) occurred when gene expression in CR or RC was not significantly different from one parent but significantly lower than the other parental line. Gene expression in CR or RC that was significantly higher than the paternal and maternal lines was regarded as over-dominance (V, VI, and VIII). Gene expression in CR or RC that was significantly lower than the paternal and maternal lines was viewed as under-dominance (III, VII, and X).

### 2.5. KEGG Pathway Analyses

The functions of the novel genes were annotated based on the Pfam database (v.31.0) [27]. To study the biological function of non-additive genes, Kyoto Encyclopedia of Genes and Genomes (KEGG) pathway analysis was performed using the Clusterprofiler package [28] in the R program. KEGG pathways with an FDR of <0.05 (BH multiple test correction) were considered significant.

### 2.6. Real-Time PCR Analysis

Total RNA was reverse-transcribed into first-strand cDNA using EasyScript First-Strand cDNA Synthesis SuperMix (TransGen, Beijng, China) for target gene detection. The primers used for qPCR detection are listed in Table S2. Each reaction was performed in a final volume of 15 μL with 1 μL of cDNA, 0.2 μL of each primer (100 nM), and 1 × PCR Mix (Power SYBR Green PCR Master Mix, Applied Biosystems, Foster City, CA, USA). Each individual sample was run in triplicate. The qPCR reactions were performed using an ABI 7500 system (Applied Biosystems, Foster City, CA, USA) with the following program: 95 °C for 10 min and 40 cycles of 95 °C for 10 s, 57 °C for 20 s, and 72 °C for 1 min. The relative expression of target genes was calculated with reference to the expression of *GAPDH*. The results are described as the fold change determined by the 2^−ΔΔCt^ method and expressed as the mean ± standard deviation (SD).

## 3. Results

### 3.1. Heterosis of Abdominal Fat Percentage in Chickens

**Figure 1:**
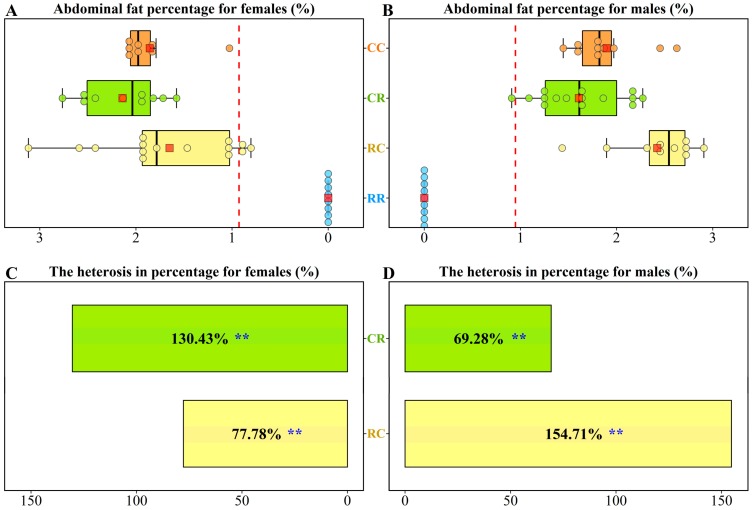
Boxplots for the abdominal fat percentages of the four groups: (**A**) females, (**B**) males. Orange, green, yellow, and blue boxes represent the CC, CR, RC, and RR groups, respectively. Each dot represents an individual. The central red point in each box represents the average value in the corresponding group. The red dashed line represents the average of the two parental lines (termed the mid-parent value). Heterosis of abdominal fat percentages for reciprocal crosses: (**C**) females, (**D**) males. Green and yellow boxes represent the CR and RC groups, respectively. The values of heterosis for the CR and RC groups are presented in the corresponding boxes. Significance was analyzed by Student’s t-test and ** indicates that *p*-value  is <0.01.

All progenies were reared in separate cages in the same environment with free access to feed and water. In consideration of the fact that modern broilers are mostly marketed at six weeks of age, 85 chickens from four groups were randomly slaughtered for carcass composition analysis at the end of six weeks of age. The AFP values of females in the CC, CR, RC, and RR groups were 1.86% ± 0.35%, 2.14% ± 0.40%, 1.65% ± 0.70%, and 0% ± 0.00%, respectively (Figure 1A). The AFP values of males in the CC, CR, RC, and RR groups were 1.90% ± 0.37%, 1.61% ± 0.43%, 2.42% ± 0.45%, and 0% ± 0.00%, respectively (Figure 1B). The AFP values of CR and RC were higher than the means of CC and RR. Heterosis as a percentage for AFP was 130.43% (*p* < 0.01) for CR females and 77.78% (*p* < 0.01) for RC females (Figure 1C). Positive heterosis for AFP was also observed in CR and RC males (Figure 1D).

### 3.2. Divergence of Gene Expression between Reciprocal Crosses and Parental Lines

**Figure 2:**
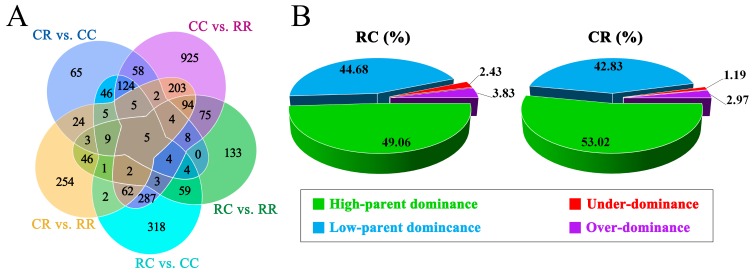
Analysis of gene expression patterns. (**A**) The number of differentially expressed genes (DEGs) among F1 progenies, and (**B**) the proportions of high-/low-parent dominant and over-/under-dominant genes in non-additive genes.

Twenty three RNA sequencing libraries from livers were constructed and sequenced. After quality control and mapping, over 100 million high-quality reads were aligned to the chicken reference genome. For each sample, over 80% of the clean reads were uniquely mapped to the reference genome. Approximately 78%, 7%, and 15% of the high-quality reads for each sample were assigned to exons, introns, and intergenic regions, respectively (Table S3). We performed principal component analysis (PCA), which revealed that the four groups were well separated in gene expression (Figure S2). The numbers of DEGs were 1861 (RR vs. CC), 366 (CR vs. CC), 936 (RC vs. CC), 450 (RC vs. RR), and 721 (CR vs. RR) (Figure 2A). These DEGs were divided into 12 types (I, II, III, IV, V, VI, VII, VIII, IX, X, XI, and XII). The number of DEGs within the 12 types were 577, 402, 0, 225, 3, 1, 28, 45, 136, 3, 435, and 608 in RC, respectively, and 717, 355, 1, 181, 0, 0, 11, 30, 128, 0, 305, and 587 in CR, respectively (Table S4). The 12 types were further classified into five main categories, namely, additivity (I, XII), high-parent dominance (II and IV), low-parent dominance (IX and XI), over-dominance (V, VI, and VIII), and under-dominance (III, VII, and X). High-/low-parent dominance and over-/under-dominance were considered non-additivity. The numbers of high-/low-parent dominant and over-/under-dominant genes in the RC group were 627, 571, 49, and 31, respectively. Further, 536, 433, 30, and 12, high-/low-parent dominant and over-/under-dominant genes were detected in the CR group, respectively. High-/low-parent dominant and over-/under-dominant genes in the RC group accounted for 49.06%, 44.68%, 2.43%, and 3.83% of non-additive genes, respectively.High-/low-parent dominant and over-/under-dominant genes in the CR group accounted for 53.02%, 42.83%, 2.97%, and 1.19% of non-additive genes, respectively (Figure 2B).

### 3.3. Functional Analysis of Non-Additive Genes

**Figure 3:**
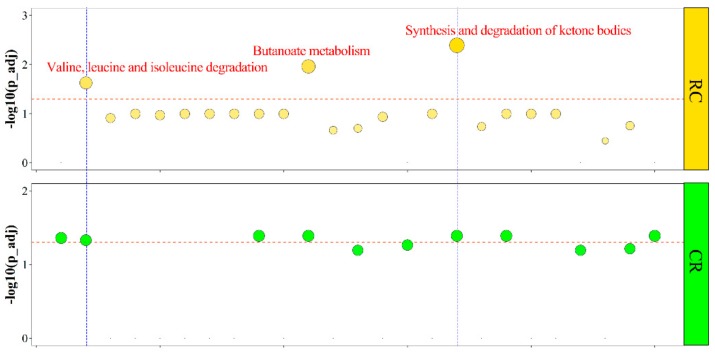
Kyoto Encyclopedia of Genes and Genomes (KEGG) pathway analysis of over-dominant genes for the RC and CR groups. Each KEGG pathway is represented by a dot. The area of a dot represents the number of genes enriched in the pathway. The dashed orange lines represent significant levels (adjusted *p*-value < 0.05). Dots that pass the blue dashed line represent significant KEGG pathways. Descriptions of the significant pathway are displayed above the dots.

We found that three lipid metabolism-related pathways—butanoate metabolism, synthesis and degradation of ketone bodies, and valine, leucine, and isoleucine degradation—were significantly enriched in over-dominant genes for both the RC and CR groups (Figure 3 and Table S5). Although steroid biosynthesis and ribosome pathways were significantly identified in high-parent dominant genes for the RC and CR groups, respectively (Figure S3A,B), no shared pathway was identified for high-parent dominant genes in the RC and CR groups. Meanwhile, no shared pathway was identified in low-parent dominant and under-dominant genes for the RC and CR groups (Figure S3C–F), despite cell cycle, DNA replication, p53 signaling, and oocyte meiosis pathways being significantly enriched in low-parent dominant genes for the CR group (Figure S3D). Three pathways, namely those associated with cholesterol metabolism, glycerolipid metabolism, and PPAR signaling, were significantly enriched in under-dominant genes for the CR group (Figure S3F).

### 3.4. Quantitative RT-PCR Validation for RNA Sequencing Data

**Figure 4:**
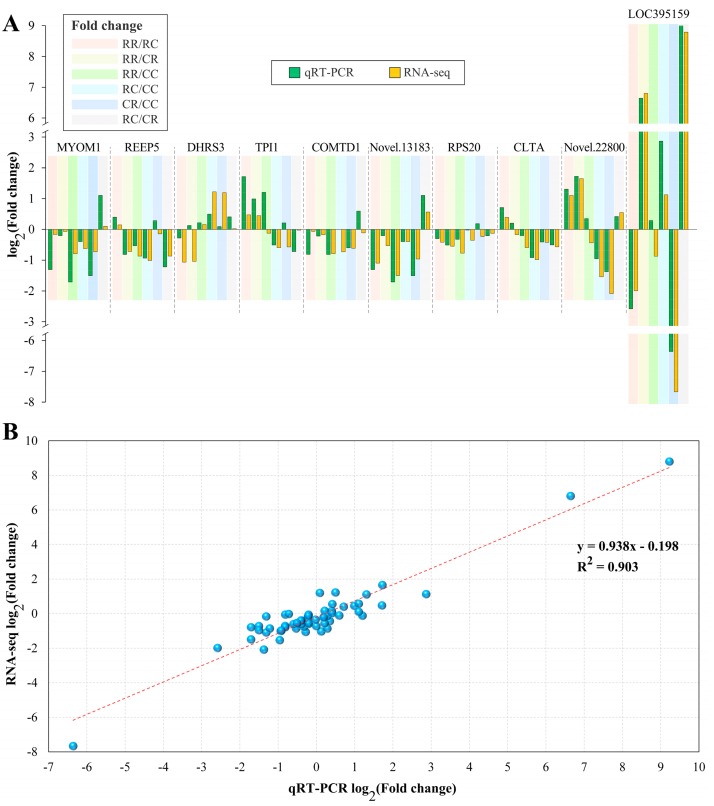
qRT-PCR validation of differentially expressed genes (DEGs) in four groups. (**A**) Comparison of log_2_(fold change) in ten DEGs between qRT-PCR and RNA-seq, and (**B**) regression analysis of log_2_(fold change) values between qRT-PCR and RNA-seq. A high R^2^ indicated that the RNA-seq data were considered to be of a high accuracy.

To validate the RNA sequencing results, we randomly detected 10 DEGs within four groups, which included up- and down-regulated genes between any two of the four groups, using quantitative reverse transcription PCR (qRT-PCR). The gene names and primer sequences are shown in Table S2. More than 85% of the DEGs between any two of the four groups of qPCR results showed the same up- and down-regulation trends as the RNA sequencing results (Figure 4A). A high R^2^ of the gene expression level was detected between the RNA sequencing and qPCR results (R^2^ = 0.90), indicating that the RNA-seq data obtained in this study were reliable (Figure 4B).

### 3.5. Gene Expression Levels of Over-Dominant Genes

**Figure 5:**
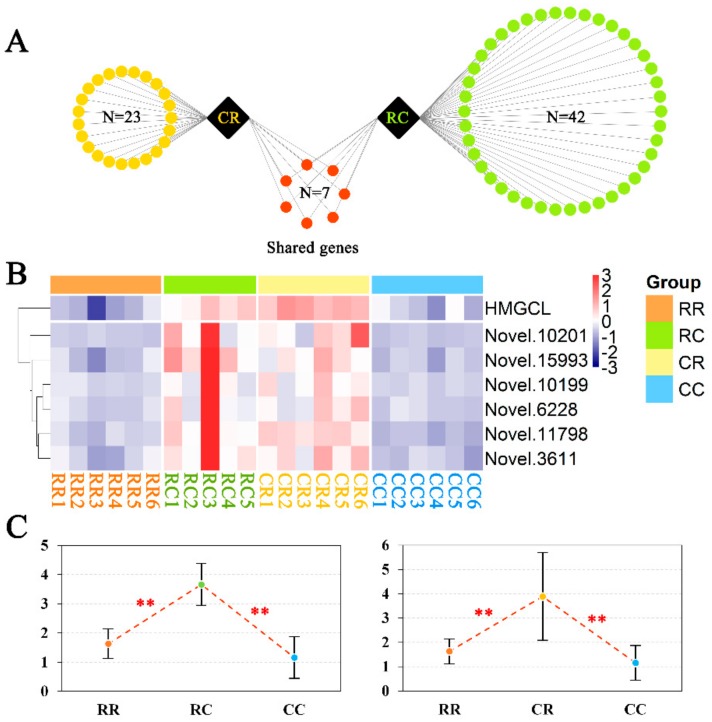
Analysis of over-dominant genes for the CR and RC groups. (**A**) Statistical analysis of the number of over-dominant genes for the CR and RC groups. Each yellow or green dot represents an over-dominant gene for CR or RC, respectively. Red dots represent the shared over-dominant genes for the CR and RC groups. (**B**) Gene expression levels of shared over-dominance for the four groups. The orange, green, yellow, and blue rectangles above the graph represent the RR, RC, CR, and CC groups, respectively. The names of the shared over-dominant genes are displayed on the right-hand side of the graph. Genes with high and low expression levels are displayed in red and blue, respectively. (**C**) qRT-PCR validated the gene expression modes of over-dominance for *HMGCL*. Because *HMGCL* was the shared gene of the CR and RC groups, which was significantly enriched in pathways of butanoate metabolism; synthesis and degradation of ketone bodies; and valine, leucine, and isoleucine degradation, we performed qRT-PCR to validate the gene expression modes. Differences between the two groups were analyzed by Student’s t-test with the SAS system. The data are expressed as the mean ± SD. * *p* < 0.05; ** *p* < 0.01.

Given that the pathways of butanoate metabolism, synthesis and degradation of ketone bodies, and valine, leucine, and isoleucine degradation were significantly enriched in over-dominant genes for both the CR and RC groups, we focused on genes exhibiting over-dominance in the CR and RC groups and found that the number of over-dominant genes was 30 in the CR group and 49 in the RC group (Table S6). Among these over-dominant genes, there were seven shared over-dominant genes (Figure 5A and Table S7). Gene cluster analysis showed that seven over-dominant genes were highly expressed in the CR and RC groups and expressed at much lower levels in the CC and RR groups (Figure 5B). Among the seven over-dominant genes, *HMGCL* was significantly enriched in the three aforementioned pathways. We further detected the gene expression levels of *HMGCL* within the four groups by qRT-PCR, validating it as an over-dominant gene (Figure 5C).

## 4. Discussion

Heterosis is of great importance for agriculture and is widely exploited in plant and animal breeding. However, the genetic mechanisms underlying this phenomenon remain elusive. Many studies have revealed that divergent patterns of gene expression between F1 crosses and their parents play significant roles in heterosis. In order to eliminate the interference of cytoplasmic inheritance, broilers and layers were used as parents to produce pure progenies and reciprocal crosses. Genome-wide transcript profiles of livers were applied to gain a global view of gene expression in the reciprocal crosses and parental lines to reveal the genetic basis of heterosis for AFP in chickens. We found that over-dominant and high-parent dominant genes were of great significance in heterosis.

In our study, both females and males exhibited great heterosis of abdominal fat deposition. Similar results were reported by Sun et al. [9], Abasht et al. [10], and Sutherland et al. [11]. Modern broilers are mostly marketed at six weeks of age. In birds, the allosomes of males are ZZ, and those of females are ZW [29], which could provide more genetic information. The liver is the major tissue site for fat deposition and metabolism in chickens. Therefore, the livers of females obtained at six weeks of age were used for RNA sequencing. A large number of DEGs were identified between reciprocal crosses and parental lines, indicating that heterosis is a consequence of polygenic effects; this was consistent with results given by previous studies [30,31]. 

Over-dominant genes accounted for a small amount of non-additive genes, while over-dominant genes of reciprocal crosses were significantly annotated by the KEGG pathways of butanoate metabolism, synthesis and degradation of ketone bodies, and valine, leucine, and isoleucine degradation, which have been reported to be associated with lipid metabolism. Our results suggest that over-dominant genes might contribute to heterosis specifically by biological function, as opposed to the number of genes. Butyrate metabolism increases lipid synthesis via the β-hydroxy-β-methylglutaryl–CoA pathway, which potentially contributes to obesity [32]. In vitro, butyrate was found to stimulate adipogenesis and triglyceride storage [33]. Ketogenic substrates are mainly derived from fatty acids, and about 4% of ketone bodies are generated from the catabolism of amino acids, particularly leucine [34]. One of the three ketone body metabolic pathways is diversion to lipogenesis or sterol synthesis in the brain, lactating mammary glands, and adipose tissue [35]. Moreover, some experimental findings demonstrated that ketone bodies contributed to about half of newly synthesized lipids and up to 75% of newly synthesized cholesterol [36,37]. Leucine, isoleucine, and valine metabolism is strongly regulated during adipogenesis [38]. 3-hydroxy-3-methylglutaryl-CoA, a leucine degradation product, is one of the intermediates of cholesterol synthesis [39]. Deficiency of isoleucine was shown to reduce sterol and fatty acid de novo synthesis in cells [40]. Since over-dominant genes of both reciprocal crosses were enriched in these three important pathways, we further analyzed the over-dominant genes of the reciprocal crosses and found seven shared over-dominant genes. Among these, *HMGCL* [41] and four novel genes annotated in the carboxylesterase family [42,43], FOXP coiled-coil domain [44,45], bZIP transcription factor [46,47,48], and reverse transcriptase [49], respectively, were reported to be associated with lipid metabolism. Interestingly, *HMGCL* was a shared over-dominant gene in the reciprocal crosses, which was annotated by KEGG pathways of butanoate metabolism, synthesis and degradation of ketone bodies, and valine, leucine, and isoleucine degradation, indicating that *HMGCL* plays important roles in heterosis. Previous research reported that 3-hydroxy-3-methylglutaryl-coenzyme A2 lyase, an essential enzyme in ketogenesis, plays important roles in lipid metabolism [50]. Genistein treatment for chickens significantly upregulated transcriptional levels of *HMGCL*, which promoted ketone body formation and cholesterol synthesis [51]. Moreover, *HMGCL* was validated as over-dominant by qRT-PCR.

The major gene expression pattern was high-parent dominance, which accounted for approximately 50% of non-additive genes in reciprocal crosses. It seems that high-parent dominant genes play important roles in heterosis. A previous study showed that high-parent dominance was important in the heterosis of *Drosophila melanogaster*, which contributed specifically to body weight heterosis [52]. A pathway of steroid biosynthesis was significantly enriched in high-parent dominant genes for the RC group. Mu et al. [30] reported that steroid biosynthesis gene up-regulation increased fat deposition in ovariectomized chickens. However, in our study, no shared significant KEGG pathway was enriched in the high-parent dominant genes of reciprocal crosses. In consideration of the fact that the pathway associated with lipid metabolism was significantly identified in high-parent dominant genes for the RC group, we could not exclude the effects of high-parent dominance expression patterns.

Chicken production has a long history of crossing different breeds to combine different economic characteristics. Heterosis of fat deposition is an unfavorable trait in chickens, which is recognized as the main source of waste in poultry production [12]. Excessive fat deposition results in consumer dissatisfaction and affects product sales [53]. Our results revealed that non-additive genes were related to lipid metabolism, particularly over-dominant genes. These findings may help in the development of effective therapies for the reduction of the heterosis of abdominal fat deposition. Our research focused on gene expression analysis for the heterosis of abdominal fat deposition in females. Further experiments should be performed to explore the genetic basis for males. 

## 5. Conclusions

In summary, this study revealed that over-dominant genes that are enriched in pathways related to lipid metabolism play important roles regarding the heterosis of fat deposition in chickens. The results of our study also showed that high-parent dominance gene expression was the typical expression pattern related to heterosis in chickens. The effects of high-parent dominant genes should not be ignored. These findings provide new insights into the genetic mechanisms of heterosis for abdominal fat deposition in chickens.

## Supplementary Materials

The following are available online at https://www.mdpi.com/2073-4425/10/10/824/s1. Figure S1: Pictures of chickens used in this experiment; Figure S2: Principal component analysis (PCA) for the four groups; Figure S3: KEGG pathway analysis of high-/low-parent dominant and under-dominant genes for RC and CR groups: (**A**,**B**) high-parent dominant genes, (**C**,**D**) low-parent dominant genes, and (**E**,**F**) under-dominant genes; Table S1: Classification of gene expression patterns in reciprocal crosses; Table S2: Primers for genes used in qRT-PCR; Table S3: Summary statistics for transcriptome sequencing data; Table S4: Summary statistics for gene expression patterns in reciprocal crosses; Table S5: KEGG pathway analysis of over-dominant genes for the RC and CR groups: (**A**) RC group, (**B**) CR group; Table S6: Detailed information of over-dominant genes in the RC and CR groups: (**A**) RC group, (**B**) CR group; Table S7: Detailed information of overlaps for over-dominant genes between the RC and CR groups.

## References

[1] Birchler J.A., Yao H., Chudalayandi S., Vaiman D., Veitia R. (2010). Heterosis. Plant Cell.

[2] Denning G., Kabambe P., Sanchez P., Malik A., Flor R., Harawa R., Nkhoma P., Zamba C., Banda C., Magombo C. (2009). Input subsidies to improve smallholder maize productivity in Malawi: Toward an African green revolution. PLoS Biol..

[3] Hara H., Hanzawa K., Yoshida Y., Watanabe S. (2013). Characterization of a chicken × peahen intergeneric hybrid produced under natural mating. Hayvansal Uretim.

[4] Springer N., Stupar R. (2007). Allelic variation and heterosis in maize: How do two halves make more than a whole?. Genome Res..

[5] Zhang Y., Ni Z., Yao Y., Nie X., Sun Q. (2007). Gibberellins and heterosis of plant height in wheat (*Triticum aestivum* L.). BMC Genet..

[6] Rahmathulla V. (2012). Management of climatic factors for successful silkworm (*Bombyx mori* L.) crop and higher silk production: A review. Psyche J. Entomol..

[7] Eeva T., Lehikoinen E. (1995). Egg shell quality, cluth size and hatching success of the great tit (*Parus major*) and the pied flycatcher (*Ficedula hypoleuca*) in an air pollution gradient. Oecologia.

[8] Havenstein G., Ferket P., Qureshi M. (2003). Growth, livability, and feed conversion of 1957 versus 2001 broilers when fed representative 1957 and 2001 broiler diets. Poult. Sci..

[9] Sun D., Wang D., Zhang Y., Yu Y., Xu G., Li J. (2005). Differential gene expression in liver of inbred chickens and their hybrid offspring. Anim. Genet..

[10] Abasht B., Lamont S. (2007). Genome-wide association analysis reveals cryptic alleles as an important factor in heterosis for fatness in chicken F2 population. Anim. Genet..

[11] Sutherland D., Honaker C., Dorshorst B., Andersson L., Siegel P. (2018). Asymmetries, heterosis, and phenotypic profiles of red junglefowl, White Plymouth Rocks, and F1 and F2 reciprocal crosses. J. Appl. Genet..

[12] Wen C., Yan W., Sun C., Ji C., Zhou Q., Zhang D., Zheng J., Ning Y. (2019). The gut microbiota is largely independent of host genetics in regulating fat deposition in chickens. ISME J..

[13] Nayeri S., Stothard P. (2016). Tissues, metabolic pathways and genes of key importance in lactating dairy cattle. Springer Sci. Rev..

[14] Fouad A., El-Senousey H. (2014). Nutritional factors affecting abdominal fat deposition in poultry: A Review. Asian Australas. J. Anim. Sci..

[15] Li L., Lu K., Chen Z., Mu T., Hu Z., Li X. (2008). Dominance, overdominance and epistasis condition the heterosis in two heterotic rice hybrids. Genetics.

[16] Song G., Guo Z., Liu Z., Cheng Q., Qu X., Chen R., Jiang D., Liu C., Wang W., Sun Y. (2013). Global RNA sequencing reveals that genotype-dependent allele-specific expression contributes to differential expression in rice F1 hybrids. BMC Plant Biol..

[17] Seymour D., Chae E., Grimm D., Martín Pizarro C., Habring-Müller A., Vasseur F., Rakitsch B., Borgwardt K., Koenig D., Weigel D. (2016). Genetic architecture of nonadditive inheritance in *Arabidopsis thaliana* hybrids. Proc. Natl. Acad. Sci. USA.

[18] Birchler J., Yao H., Chudalayandi S. (2006). Unraveling the genetic basis of hybrid vigor. Proc. Natl. Acad. Sci. USA.

[19] Swanson-Wagner R., Jia Y., DeCook R., Borsuk L., Nettleton D., Schnable P. (2006). All possible modes of gene action are observed in a global comparison of gene expression in a maize F1 hybrid and its inbred parents. Proc. Natl. Acad. Sci. USA.

[20] Gu H., Qi X., Jia Y., Zhang Z., Nie C., Li X., Li J., Jiang Z., Wang Q., Qu L. (2019). Inheritance patterns of the transcriptome in hybrid chickens and their parents revealed by expression analysis. Sci. Rep..

[21] Zhuo Z., Lamont S., Abasht B. (2019). RNA-Seq analyses identify additivity as the predominant gene expression pattern in F1 chicken embryonic brain and liver. Genes.

[22] Wu Z., Zhang C. (1983). Heterosis and statistical tests. Hereditas.

[23] Kim D., Langmead B., Salzberg S. (2015). HISAT: A fast spliced aligner with low memory requirements. Nat. Methods.

[24] Pertea M., Pertea G., Antonescu C.M., Chang T., Mendell J., Salzberg S. (2015). StringTie enables improved reconstruction of a transcriptome from RNA-seq reads. Nat. Biotechnol..

[25] Love M., Huber W., Anders S. (2014). Moderated estimation of fold change and dispersion for RNA-seq data with DESeq2. Genome Biol..

[26] Rapp R., Udall J., Wendel J. (2009). Genomic expression dominance in allopolyploids. BMC Biol..

[27] El-Gebali S., Mistry J., Bateman A., Eddy S., Luciani A., Potter S., Qureshi M., Richardson L., Salazar G., Smart A. (2019). The Pfam protein families database in 2019. Nucleic Acids Res..

[28] Yu G., Wang L., Han Y., He Q. (2012). clusterProfiler: An R package for comparing biological themes among gene clusters. Omics J. Integr. Biol..

[29] Schoenmakers S., Wassenaar E., Hoogerbrugge J., Laven J., Grootegoed J., Baarends W. (2009). Female meiotic sex chromosome inactivation in chicken. PLoS Genet..

[30] Wu X., Li R., Li Q., Bao H., Wu C. (2016). Comparative transcriptome analysis among parental inbred and crosses reveals the role of dominance gene expression in heterosis in *Drosophila melanogaster*. Sci. Rep..

[31] Wang H., Fang Y., Wang L., Zhu W., Ji H., Wang H., Xu S., Sima Y. (2016). Heterosis and differential gene expression in hybrids and parents in *Bombyx mori* by digital gene expression profiling. Sci. Rep..

[32] Liu H., Wang J., He T., Becker S., Zhang G., Li D., Ma X. (2018). Butyrate: A double-edged sword for health?. Adv. Nutr..

[33] Yan H., Ajuwon K. (2015). Mechanism of butyrate stimulation of triglyceride storage and adipokine expression during adipogenic differentiation of porcine stromovascular cells. PLoS ONE.

[34] Thomas L., Ittmann M., Cooper C. (1982). The role of leucine in ketogenesis in starved rats. Biochem. J..

[35] Puchalska P., Crawford P. (2017). Multi-dimensional roles of ketone bodies in fuel metabolism, signaling, and therapeutics. Cell Metab..

[36] Geelen M., Lopes-Cardozo M., Edmond J. (1983). Acetoacetate: A major substrate for the synthesis of cholesterol and fatty acids by isolated rat hepatocytes. FEBS Lett..

[37] Freed L., Endemann G., Tomera J., Gavino V., Brunengraber H. (1988). Lipogenesis from ketone bodies in perfused livers from streptozocin-induced diabetic rats. Diabetes.

[38] Du Y., Meng Q., Zhang Q., Guo F. (2012). Isoleucine or valine deprivation stimulates fat loss via increasing energy expenditure and regulating lipid metabolism in WAT. Amino Acids.

[39] Halama A., Horsch M., Kastenmüller G., Möller G., Kumar P., Prehn C., Laumen H., Hauner H., Hrabĕde Angelis M., Beckers J. (2016). Metabolic switch during adipogenesis: From branched chain amino acid catabolism to lipid synthesis. Arch. Biochem. Biophys..

[40] Maltest W., Reitz B., Volpe J. (1981). Effects of isoleucine deprivation on synthesis of sterols and fatty acids in LM-cells. J. Biol. Chem..

[41] Alam M., Rahman M. (2014). Mitochondrial dysfunction in obesity: Potential benefit and mechanism of Co-enzyme Q10 supplementation in metabolic syndrome. J. Diabetes Metab. Disord..

[42] Ruby M., Massart J., Hunerdosse D., Schönke M., Correia J., Louie S., Ruas J., Näslund E., Nomura D., Zierath J. (2017). Human carboxylesterase 2 reverses obesity-induced diacylglycerol accumulation and glucose intolerance. Cell Rep..

[43] Jones R., Taylor A., Tong E., Repa J. (2012). Carboxylesterases are uniquely expressed among tissues and regulated by nuclear hormone receptors in the mouse. Drug Metab. Dispos..

[44] Howie D., Cobbold S., Adams E., Ten B., Necula A., Zhang W., Huang H., Roberts D., Thomas B., Hester S. (2017). Foxp3 drives oxidative phosphorylation and protection from lipotoxicity. JCI Insight.

[45] Golson M., Kaestner K. (2016). Fox transcription factors: From development to disease. Development.

[46] Kwon S., Kang N., Koh H., Shin S., Lee B., Jeong B., Chang Y. (2018). Enhancement of biomass and lipid productivity by overexpression of a bZIP transcription factor in *Nannochloropsis salina*. Biotechnol. Bioeng..

[47] Yamaoka Y., Shin S., Choi B., Kim H., Jang S., Kajikawa M., Yamano T., Kong F., Légeret B., Fukuzawa H. (2019). The bZIP1 transcription factor regulates lipid remodeling and contributes to ER stress management in *Chlamydomonas reinhardtii*. Plant Cell..

[48] Li D., Balamurugan S., Yang Y., Zheng J., Huang D., Zou L., Yang W., Liu J., Guan Y., Li H. (2019). Transcriptional regulation of microalgae for concurrent lipid overproduction and secretion. Sci. Adv..

[49] Sension M., Deckx H. (2015). Lipid metabolism and lipodystrophy in HIV-1-infected patients: The role played by nonnucleoside reverse transcriptase inhibitors. AIDS Rev..

[50] Luo W., Qin L., Li B., Liao Z., Liang J., Xiao X., Xiao X., Mo Y., Huang G., Zhang Z. (2017). Inactivation of HMGCL promotes proliferation and metastasis of nasopharyngeal carcinoma by suppressing oxidative stress. Sci. Rep..

[51] Lv Z., Fan H., Song B., Li G., Liu D., Guo Y. (2019). Supplementing genistein for breeder hens alters the fatty acid metabolism and growth performance of offsprings by epigenetic modification. Oxid. Med. Cell. Longev..

[52] Mu X., Cui X., Liu R., Li Q., Zheng M., Zhao G., Ge C., Wen J., Hu Y., Cui H. (2019). Identification of differentially expressed genes and pathways for abdominal fat deposition in ovariectomized and sham-operated chickens. Genes.

[53] Wen C., Yan W., Zheng J., Ji C., Zhang D., Sun C., Yang N. (2018). Feed efficiency measures and their relationships with production and meat quality traits in slower growing broilers. Poult. Sci..

