# Supplementary figures and images for: Implications of Gene Inheritance Patterns on the Heterosis of Abdominal Fat Deposition in Chickens
Source: Genes (Basel). 2019 Oct 18;10(10):824. doi: 10.3390/genes10100824 (PMC6826362; doi:10.3390/genes10100824)

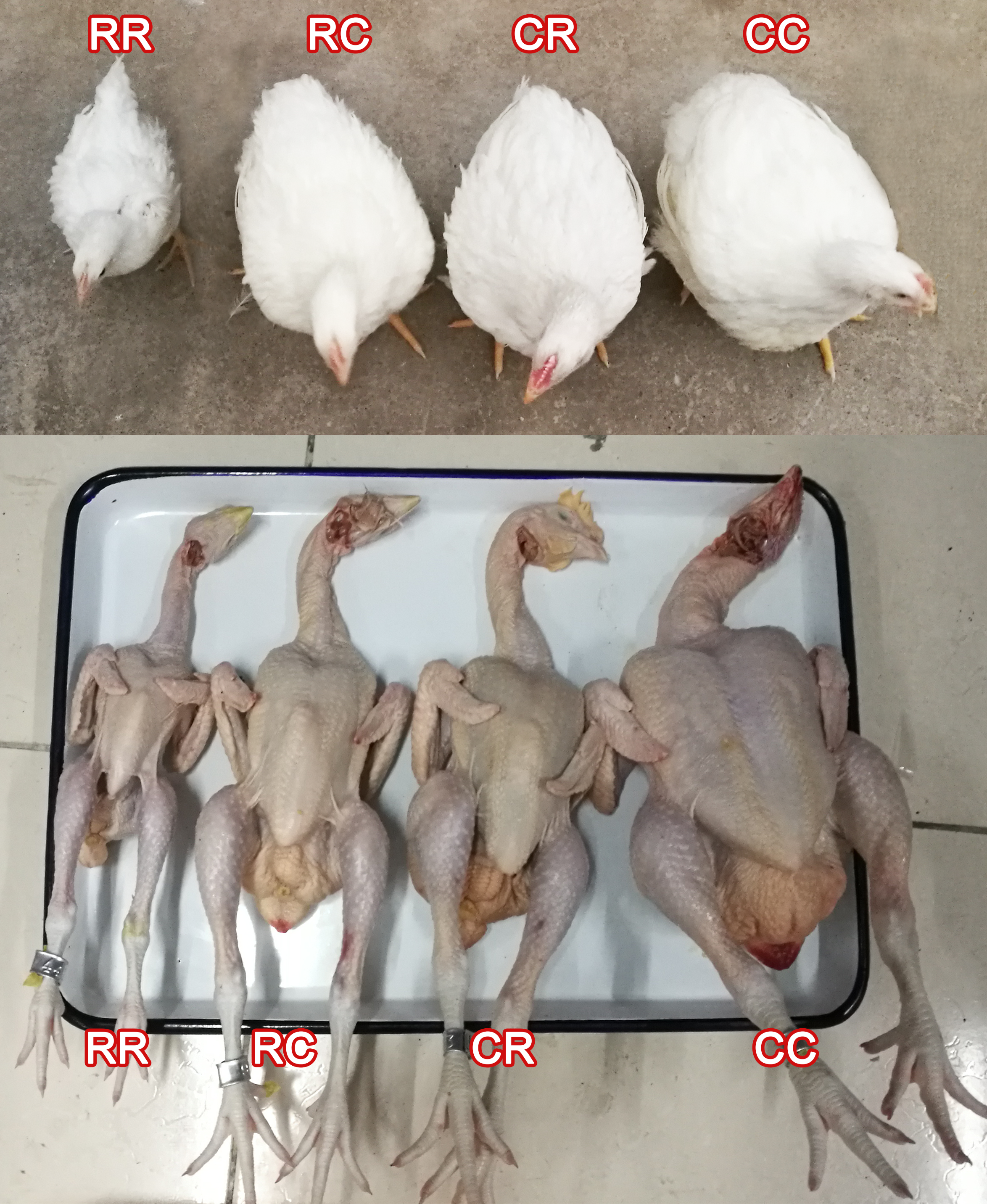

Supplement: Supplementary file 1 [file genes-10-00824-s001.zip › supplementary files/FigureS1.tif]

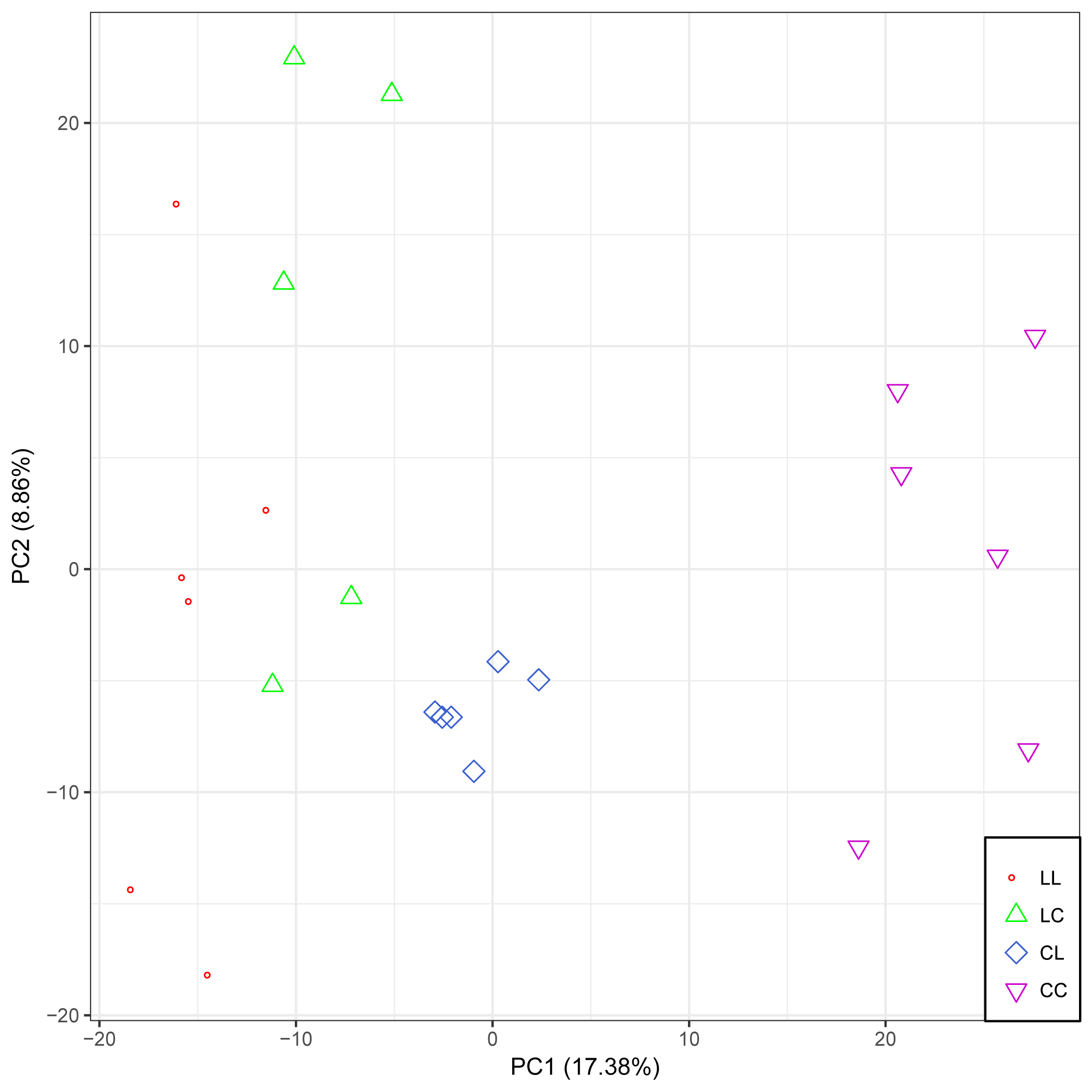

Supplement: Supplementary file 1 [file genes-10-00824-s001.zip › supplementary files/FigureS2.tif]

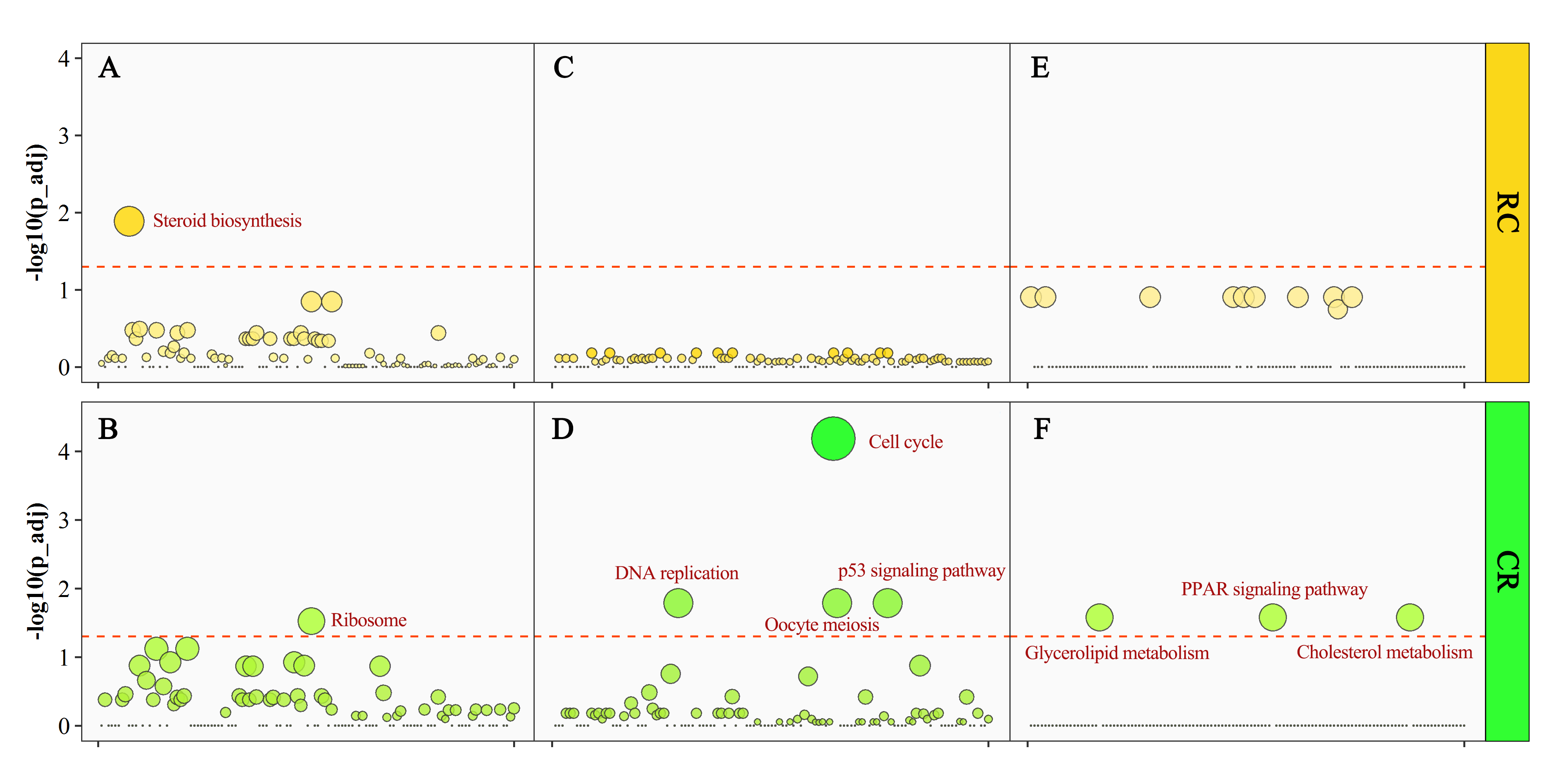

Supplement: Supplementary file 1 [file genes-10-00824-s001.zip › supplementary files/FigureS3.tif]
